# MicroRNA Dysregulation in HPV-Driven Cervical Cancer: A Review of Oncoprotein-Targeted Signaling Pathways

**DOI:** 10.3390/life16081332

**Published:** 2026-08-14

**Authors:** Geovanna Marques Pereira, Stéphanie Calfa, Pedro Rassier dos Santos, Ana Julia Aguiar de Freitas, Stefanne Maria Jeha Bortoletto, Rui Manuel Reis, Márcia Maria Chiquitelli Marques, Rhafaela Lima Causin

**Affiliations:** 1 Molecular Oncology Research Center, Teaching and Research Institute, Barretos Cancer Hospital, Barretos 14784-400, SP, Brazil; geovannam0529@gmail.com (G.M.P.); stephaniecalfa@outlook.com (S.C.); rassier1907@gmail.com (P.R.d.S.); aaguiardefreitas@gmail.com (A.J.A.d.F.); stefannebortoletto@gmail.com (S.M.J.B.); ruireis.hcb@gmail.com (R.M.R.); mmcmsilveira@gmail.com (M.M.C.M.); 2 Life and Health Sciences Research Institute (ICVS), Medical School, University of Minho, 4710-057 Braga, Portugal

**Keywords:** cervical cancer, microRNAs, HPV, signaling pathways, hallmarks of cancer

## Abstract

MicroRNAs (miRNAs) have been associated with the initiation, development, and progression of various cancers, including cervical cancer. Their involvement in cervical cancer is extensively documented, as they influence critical biological pathways, including apoptosis, cell cycle progression, immune evasion, and metastasis. In cervical cancer, deregulated miRNA expression contributes to tumor aggressiveness by interfering with key molecular pathways, many of which are also influenced by high-risk human papillomavirus (HPV) oncoproteins. In this review, we highlight key signaling pathways regulated by miRNAs linked to cancer hallmarks, particularly sustained proliferative signaling, which was the most frequently affected pathway across the studies reviewed. Furthermore, the interplay among HPV oncoproteins, dysregulated miRNA expression, and altered signaling pathways drives key oncogenic processes, including uncontrolled proliferation, evasion of apoptosis, and metastasis.

## 1. Introduction

Cervical cancer (CC) is the fourth most common cancer and has the highest mortality rate among women worldwide [1]. In Brazil, it is estimated that 17,000 new cases will be diagnosed in the 2023–2025 triennium, with an estimated incidence rate of 15.38 cases per 100,000 women, making it the third most common cancer among women in the country [2]. Human Papillomavirus (HPV) infection is the leading etiological factor for CC [3]. HPV can be classified by oncogenic potential into low-risk (lr-HPV) and high-risk (hr-HPV) types. Lr-HPV types cause genital warts, while hr-HPV types are carcinogenic [4,5]. Persistent HPV infection can lead to the development of cervical intraepithelial neoplasia (CIN), which may progress to CC [6].

During the progression of CC, the HPV oncoproteins E6 and E7 degrade the tumor suppressor proteins p53 and retinoblastoma protein (pRb), respectively, leading to cell cycle dysregulation and uncontrolled cell proliferation, which promote cancer development [3]. These oncoproteins can also induce epigenetic mechanisms, such as DNA methylation and histone modification, contributing to tumor progression [3,6]. Epigenetic modifications and gene expression regulation are further modulated by microRNAs (miRNAs), which play a critical role in this process.

miRNAs are small, non-coding RNAs, typically 18–24 nucleotides in length, that play a crucial role in regulating gene expression. They are involved in various biological processes, including development, cell division, differentiation, apoptosis, and disease progression, including cancer [7,8]. The biogenesis, function, and regulation of miRNAs are complex and tightly controlled, as even minor disruptions in miRNA pathways can lead to significant pathological conditions, such as cancer, cardiovascular diseases, and neurodegenerative disorders [9]. In carcinogenesis, miRNAs are classified into oncogenic miRNAs (oncomiRs) or tumor-suppressor genes (ts-miRs) [9,10,11].

miRNAs are highly selective and can regulate a broad spectrum of genes, often targeting multiple mRNAs simultaneously. This ability enables miRNAs to fine-tune gene expression and modulate various biological processes, including development and differentiation, cell cycle and apoptosis, and immune response [12]. The expression of miRNAs is precisely regulated at several stages to ensure proper cellular function and to prevent pathological states [13]. Key mechanisms involved in miRNA regulation include transcriptional regulation [14], epigenetic modifications [15,16], post-transcriptional regulation [17], regulation by other RNAs [18], environmental and physiological factors, and feedback loops [19].

miRNAs play a crucial role in regulating and modulating the hallmarks of cancer, including sustaining proliferative signaling, evading growth suppressors, resisting cell death, enabling replicative immortality, and activating invasion and metastasis [20]. In CC, miRNAs influence biological pathways involved in development and progression, acting as key factors in carcinogenesis [21]. Given the significant role to understanding its molecular mechanisms. These pathways may provide valuable insights into novel biomarkers for early detection and prognosis, as well as therapeutic targets for CC. Moreover, understanding how miRNAs regulate key signaling pathways in cervical carcinogenesis could lead to the development of more effective, targeted therapies that improve patient outcomes. Therefore, the findings of this review aim to make a significant contribution to future research on CC and support the development of more effective approaches to the early diagnosis and treatment of the disease. Although several reviews have explored the role of miRNAs in CC, many studies primarily focus on their diagnostic or prognostic value; few reviews integrate the mechanistic relationships among HPV oncoproteins, miRNA dysregulation, and the modulation of specific oncogenic signaling pathways involved in CC progression. Therefore, this review aims to provide an overview of HPV-mediated miRNA dysregulation in CC, with an emphasis on signaling pathways.

## 2. Association of microRNA Expression in the Development and Progression of Cervical Cancer

Numerous miRNAs exhibit dual roles in different tumors, acting as suppressors in some cancers and activators in others, highlighting the importance of regulatory mechanisms and the complex, multitarget interactions between miRNAs and their regulators [22]. In this context, dysregulated miRNA expression contributes significantly to the evolution of precursor lesions and plays a critical role in the progression of precursor lesions and the development of CC, a complex mainly instigated by hr-HPV infection [23]. Persistent hr-HPV infection is a key initiating factor in the pathogenesis of CC. However, while necessary, it is not sufficient for malignant transformation, since additional genetic and epigenetic alterations, including changes in miRNA expression, contribute to tumor progression [24]. Several studies have demonstrated that miRNAs are key regulators of critical cellular processes, including apoptosis [25], cell cycle progression [23], and metastasis [26], all of which are vital to cancer development and progression [22].

A prominent example of miRNA involvement in CC is miRNA-10a-5p [27]. Its upregulation is linked to enhanced cell growth and migration, as well as to inhibition of the ubiquitin-conjugating enzyme E2I (UBE2I), which is a critical regulator of apoptosis and cell differentiation. Thus, a better understanding of the molecular basis of miRNA-10a-5p function could lead to new, useful avenues in clinical practice. On the other hand, modulation of the miRNA-10a-5p/UBE2I axis might be an encouraging approach to hinder the tumor spread and improve patients’ outcomes; it needs to be investigated further and clinically validated [27].

Goe et al. identified seven upregulated miRNAs (miR-15a-5p, miR-17-5p, miR-20a-5p, miR-21-5p, miR-96, miR-106b-5p, and miR-3653) associated with key oncogenic processes, including enhanced cell proliferation, inhibition of apoptosis, and HPV-driven tumor progression [28]. Furthermore, miR-21 expression increased progressively with lesion severity, supporting its potential role as an oncomiR involved in the transition from precursor lesions to invasive CC [29]. These findings suggest that coordinated dysregulation of multiple miRNAs contributes to HPV-driven carcinogenesis by promoting proliferation and reducing apoptotic responses.

A systematic review published in 2021 offered a broader perspective by examining the results of 27 studies, which identified 26 miRNAs associated with key aspects of CC progression, such as cellular proliferation, migration, and apoptosis [21]. Some specific downregulated miRNAs, such as miR-1284, miR-573, miR-433, miR-424-5p, and miR-361-5p, exhibit distinct expression patterns across various stages of CC. Their dysregulation influences hallmarks of cancer processes such as evasion of growth suppressors and induction of replicative immortality. These findings emphasize the clinical potential of miRNAs as biomarkers for disease classification, prognosis, and therapeutic intervention.

Incorporating miRNAs as molecular markers into screening protocols can enhance the accuracy of patient stratification by considering their risk profiles. This enables the adoption of personalized therapeutic approaches that not only improve clinical outcomes but also help reduce the overall impact of CC [30]. Beyond refining the predictive capacity of screening programs, understanding these relationships opens new pathways for developing more targeted therapies within precision medicine.

## 3. HPV Oncoproteins and miRNA-Mediated Signaling Pathways

**Figure 1:**
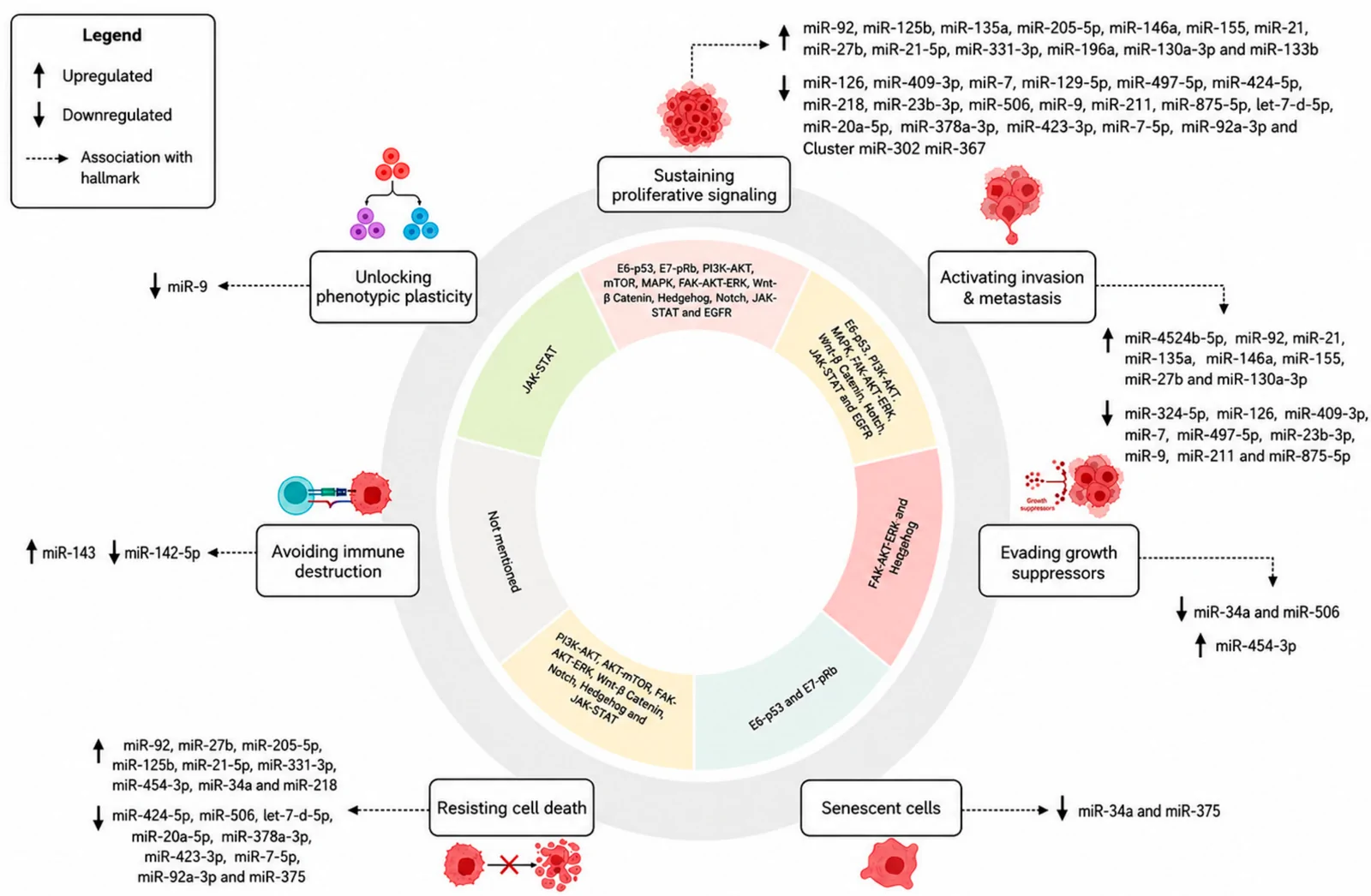
miRNAs are associated with signaling pathways and hallmarks of cancer. Upregulated miRNAs are indicated by ↑, and downregulated miRNAs are indicated by ↓. The red X in the Resisting cell death hallmark denotes inhibition of apoptosis, illustrating the ability of cancer cells to evade programmed cell death.

**Figure 2:**
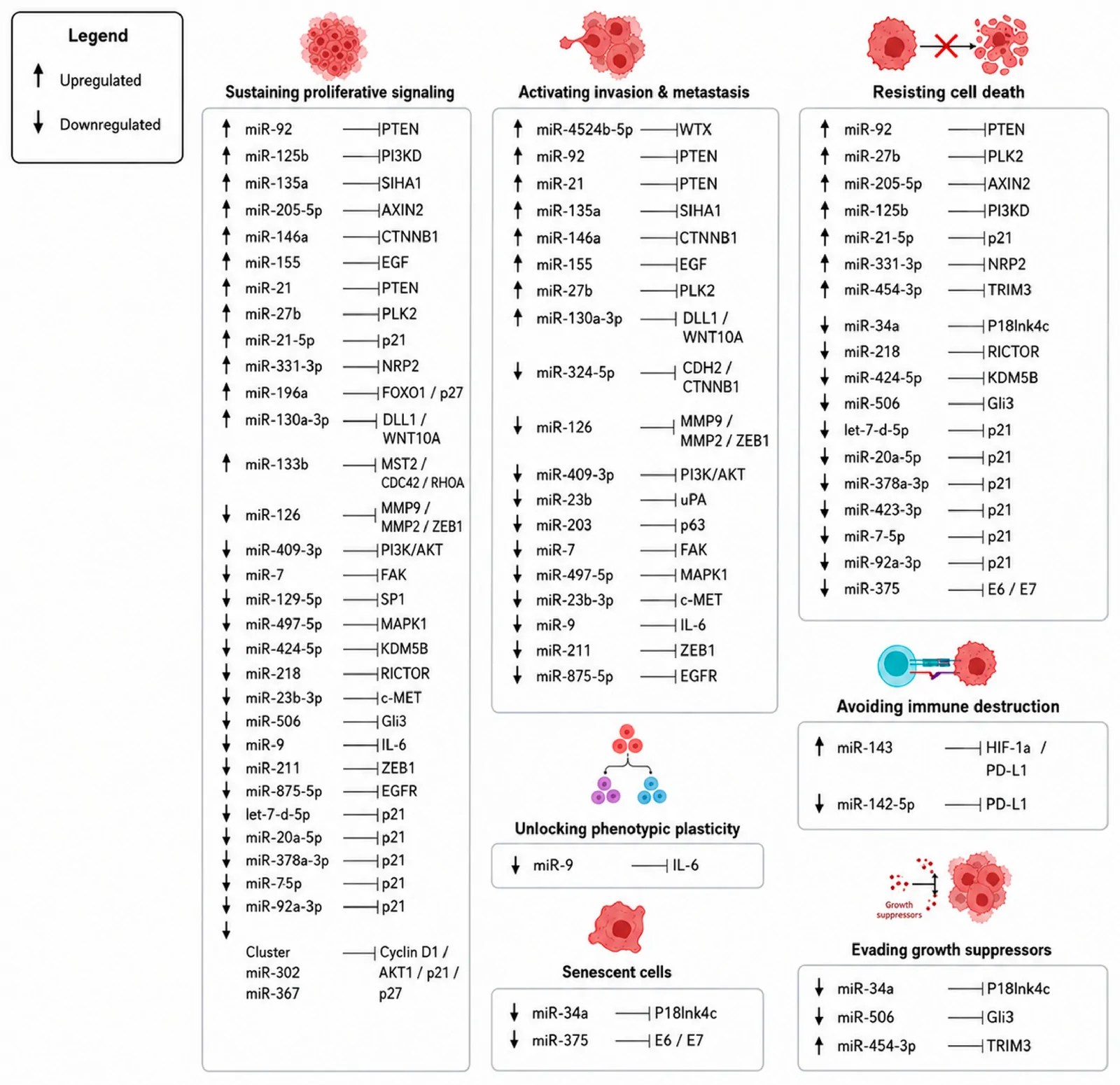
miRNAs are involved in CC. The upregulated and downregulated miRNAs associated with various hallmarks in CC are indicated by ↑ and ↓, respectively. The symbol ┤ indicates that the miRNA negatively regulates its target. The red X in the Resisting cell death hallmark denotes inhibition of apoptosis, illustrating the ability of cancer cells to evade programmed cell death.

The oncogenic potential of hr-HPV is mediated by the viral oncoproteins E5, E6, and E7, which disrupt key cellular pathways and regulate the expression of miRNAs, crucial molecules that control processes such as proliferation, migration, apoptosis, and immune evasion [25,30]. These oncoproteins work synergistically to promote hallmarks of cancer, including uncontrolled growth, resistance to cell death, and immune escape [30]. Several miRNAs exhibit aberrant expression in signaling pathways associated with HPV-driven carcinogenesis, including E6-p53, E7-pRb, PI3K/AKT, FAK/AKT/ERK, Wnt/β-catenin, Notch, Hedgehog, JAK/STAT, and EGFR pathways (Figure 1 and Figure 2) [31,32,33,34]. Such dysregulation contributes to abnormal cell proliferation and differentiation, ultimately favoring CC development [32,35]. A summary of HPV oncoprotein-associated miRNAs and their molecular targets is presented in Table 1.

The E5 oncoprotein promotes immune evasion and cell transformation by interacting with major histocompatibility complex class I (MHC I) to impair immune detection. It enhances epidermal growth factor receptor (EGFR) activation, drives cell proliferation, and helps cells evade apoptosis by disrupting the Fas receptor and the Death-Inducing Signaling Complex (DISC) [45,46].

The E5 oncoprotein can alter miRNA expression to influence cell adhesion, migration, proliferation, and Wnt signaling, contributing to CC progression [47]. Greco et al. demonstrated that HPV16-positive keratinocytes expressing E5 can downregulate miR-203 and miR-324-5, while upregulating miR-146a. The upregulation of miR-146a promotes cell adhesion and cycle progression by targeting zinc finger protein (ZNF813). Additionally, miR-146a inhibits catenin beta 1 (CTNNB1), an intermediary gene in the Wnt pathway, impairing proliferation, migration, and invasion [48].

In contrast, the downregulation of miR-203 affects cell junctions, migration, and motility by regulating p63, thereby contributing to cellular transformation and tumor progression [36]. E5 also downregulates miR-324-5p, which targets CDH2 and CTNNB1, contributing to transendothelial migration.

The E6 oncoprotein targets p53, altering miRNA expression, degrading tumor-suppressive factors, and regulating signaling pathways [49]. In CC, miR-34a is downregulated due to HPV E6-mediated degradation of p53. This negative regulation results in increased expression of p18 inhibitor of cyclin-dependent kinase 4c (P18INK4c), a cyclin-dependent kinase inhibitor, which is associated with CC progression [50,51]. Additionally, miR-34a reduces the expression of Notch1 and Jagged1, inhibiting cervical cell invasion [52]. Likewise, E6 negatively in concert with p53, regulates miR-23b, leading to increased expression of Urokinase-type plasminogen activator (uPA), which promotes cell migration [53].

The E6 oncoprotein upregulates miR-92, which in turn downregulates the expression of the phosphatase and tensin homolog (PTEN), thus activating the phosphoinositide-3 kinase (PI3K)/AKT pathway [54]. Conversely, miR-125b negatively regulates the catalytic subunit of phosphoinositide 3-kinase delta (PIK3CD), thereby suppressing tumor growth and promoting apoptosis in cancer cells. PIK3CD regulates the PI3K pathway [55]. Sommerova et al. identified several miRNAs associated with cervical carcinogenesis, highlighting the downregulation of miR-409-3p and its inverse correlation with E6 mRNA levels. E6 suppresses miR-409-3p, activating the PI3K/AKT pathway, which enhances cell proliferation and migration, suggesting a potential tumor-suppressor role for miR-409-3p [37].

In addition to proliferation-related pathways, E6 modulates immune evasion mechanisms. Knocking out E6 in CC cells resulted in increased miR-143 expression and decreased hypoxia-inducible factor 1-alpha (HIF-1α) and programmed death-ligand 1 (PD-L1) levels, suggesting that E6 promotes immune evasion via the miR-143/HIF-1α axis [38].

The E7 oncoprotein inactivates pRb, driving uncontrolled cell division [56]. Together, these oncoproteins promote tumor progression and therapy resistance. Understanding their effects on miRNAs offers insights into CC mechanisms and potential targeted therapies [57]. In addition, E7 regulates several miRNAs associated with proliferation, invasion, apoptosis resistance, and therapeutic response.

Liu et al. showed that E7 upregulates miR-27b via DiGeorge critical region 8 (DGCR8), leading to downregulation of polo-like kinase 2 (PLK2), thereby promoting CC cell proliferation and invasion while reducing paclitaxel-induced apoptosis. This E7/miR-27b/PLK2 pathway presents a potential target for CC therapy [39]. Similarly, Kong et al. described that the E7 oncoprotein upregulates miR-21, enhancing CC cell growth, proliferation, and invasion. Inhibiting miR-21 in E7-overexpressing cells reduces these effects without significantly affecting apoptosis, highlighting miR-21 as a key mediator of E7-driven CC progression [40]. miR-21 also inhibits PTEN, thus activating the AKT pathway [58]. E7-associated miRNAs are also involved in MAPK and Wnt/β-catenin signaling. Overexpression of miR-133b activates the AKT1 and extracellular signal-regulated kinase (ERK) pathway, promoting cell proliferation through the degradation of its targets, mammalian sterile 20-like kinase 2 (MST2), cell division control protein 42 homolog (CDC42), and ras homolog gene family member A (RHOA) [59]. In addition, miR-205-5p negatively regulates axis inhibition protein 2 (AXIN2), activating the Wnt/β-catenin signaling pathway, inhibiting apoptosis, and increasing cell proliferation [60].

The E6/E7 oncoproteins together can regulate various miRNAs that influence key processes such as proliferation, apoptosis, and immune evasion in CC. For example, Honegger et al. demonstrated that E6/E7 oncoproteins regulate intracellular and exosomal miRNAs in HPV-positive cancer cells, impacting key processes such as proliferation and apoptosis. Silencing E6/E7 in HeLa and SiHa cells downregulated miR-17-5p, miR-186-5p, miR-378a-3p, miR-378f, miR-629-5p, and miR-7-5p, while upregulating miR-143-3p, miR-23a-3p, miR-23b-3p, and miR-27b-3p. Additionally, in exosomes, E6/E7 downregulated let-7d-5p, miR-20a-5p, miR-378a-3p, miR-423-3p, miR-7-5p, and miR-92a-3p, while upregulating miR-21-5p, which targets p21 to promote cell proliferation and inhibit apoptosis. These findings highlight the role of E6/E7 in miRNA-mediated regulation of HPV-driven cancer growth [41].

The E6/E7 complex also modulates immune-related signaling pathways. Ling et al. showed that HPV16 E6/E7 downregulates miR-142-5p, thereby increasing PD-L1 expression and promoting immune evasion in CC. Restoring miR-142-5p inhibits tumor growth by targeting PD-L1, suggesting its potential as a therapeutic target for immunotherapy [42]. Furthermore, another study reported that HPV16 E6/E7 upregulates miR-331-3p, which targets neuropilin 2 (NRP2), thereby influencing cell proliferation, cell cycle progression, and apoptosis. NRP2 also regulates E6/E7 expression and keratinocyte differentiation markers. These findings suggest that miR-331-3p and NRP2 play key roles in CC progression and could be potential targets for therapeutic interventions [43].

The oncoproteins E5, E6, and E7 can cooperatively regulate miRNAs involved in cell proliferation and tumor growth, highlighting their potential as biomarkers for CC. Han et al. demonstrated that HPV16 oncoproteins E5, E6, and E7 collectively downregulate miR-148a-3p, which regulates cell proliferation and may contribute to tumor growth. Additionally, E6/E7 suppresses miR-199b-5p and miR-190a-5p, further promoting CC progression. Silencing these oncoproteins restores the expression of these miRNAs, highlighting their potential as diagnostic biomarkers for HPV16-positive CC [44].

Additionally, miR-375 functions as a tumor suppressor by negatively regulating the HPV E6 and E7 oncogenes, which are crucial for the degradation of p53 and pRb, respectively [61]. Suppression of E6 and E7 by miR-375 stabilizes p53, inducing cell cycle arrest and apoptosis in HPV-positive CC cells [62]. miR-129-5p negatively regulates specificity protein 1 (SP1), a transcription factor that promotes HPV E6 and E7 expression, thereby reducing the oncogenic potential of HPV in CC cells [63].

Beyond direct regulation by viral oncoproteins, several dysregulated miRNAs influence major signaling pathways involved in cervical carcinogenesis. Table 2 summarizes the major dysregulated miRNAs involved in signaling pathways associated with cervical carcinogenesis.

miR-135a regulates the Wnt/β-catenin signaling pathway via seven in absentia homolog 1 (SIAH1), promoting β-catenin degradation [71]. miR-4524b-5p downregulates Wilms tumor gene on the X chromosome (WTX), also known as APC membrane recruitment protein 1 (AMER1), which promotes the ubiquitination and degradation of β-catenin, thus negatively regulating the Wnt signaling pathway [72]. Overexpression of miR-424-5p inhibits the lysine demethylase 5B (KDM5B) expression, which suppresses Notch1 and Notch2 expression, thereby reducing the activation of the Notch pathway and decreasing cell proliferation [73]. miR-130a-3p targets delta-like notch 1 ligand (DLL1), a Notch ligand, leading to its downregulation in CC tissues. Inhibition of miR-130a-3p reduces proliferation, migration, and invasion of CC cells, suggesting its role in promoting cancer cell aggressiveness through DLL1 modulation [75]. Furthermore, a study by Causin et al. demonstrated that Wnt family member 10a (WNT10A), a member of the Wnt signaling pathway, is a target of miR-130a-3p, with reduced WNT10A expression observed upon miR-130a-3p overexpression [75].

miR-506 suppresses CC cell growth by targeting GLI family zinc finger 3 (Gli3), a transcription factor in the Hedgehog signaling pathway [76]. miR-9, a tumor suppressor, targets interleukin-6 (IL-6) in the JAK/STAT3 signaling pathway, leading to autophosphorylation and activation of JAK [77]. miR-126 inhibits the expression of matrix metalloproteinase-2 (MMP2), matrix Metalloproteinase-9 (MMP9), and the JAK2/STAT3 pathway through its target, zinc finger E-box binding homeobox 1 (ZEB1) [78]. Additionally, ZEB1 is a target of miR-211, and these two miRNAs inhibit proliferation, migration, and invasion by regulating ZEB1 [79].

The epidermal growth factor receptor (EGFR) is downregulated by miR-875-5p, which suppresses growth and metastasis in CC [80]. Moreover, the epidermal growth factor (EGF) induces EMT, which can be downregulated by miR-155. Lei et al. demonstrated that negative regulation of miR-155 inhibits proliferation, migration, and enhances chemosensitivity in CC cells [81].

The miR-302 and miR-367 cluster downregulated cyclin D1 and AKT1 through 3′-UTR interactions, indirectly upregulating p27 and p21, which inhibit cell proliferation [64]. A study by Hou et al. showed that miR-196a downregulates forkhead box O1 (FOXO1) and p27, contributing to cancer cell proliferation [65]. Li et al. demonstrated that overexpression of miR-218 in HeLa cells promotes apoptosis by targeting the AKT/mTOR signaling pathway, while also reducing cell proliferation [66].

miR-23b-3p acts as a tumor suppressor by targeting c-mesenchymal–epithelial transition factor (c-MET), promoting epithelial–mesenchymal transition (EMT), cell migration, and cancer cell invasion. Overexpression of miR-23b-3p reduces focal adhesion kinase (FAK) activation, a downstream effector of c-MET [67]. miR-7 targets FAK, leading to decreased FAK protein levels and suppression of cell proliferation, migration, and invasion [68]. Lu et al. demonstrated that miR-497-5p acts as a tumor suppressor by negatively regulating mitogen-activated protein kinase 1 (MAPK1), hence decreasing ERK activation [69]. miR-454-3p targets tripartite motif-containing 3 (TRIM3), activating P38 MAPK through downregulation by P53 and cleaved caspase-3 [70]. Additionally, miR-628-5p regulates Jagged1, activating the Notch signaling pathway [74]. Despite the well-established roles of these signaling pathways in CC progression, the complexity of their interactions necessitates further studies to elucidate their implications for therapeutic strategies fully. Understanding these mechanisms could lead to more effective cancer treatments by targeting specific nodes within these signaling networks.

## 4. Clinical Significance of Deregulation of microRNA Expression in Cervical Cancer

In CC, miRNAs can act as tumor suppressors or oncogenic factors, regulating gene expression, cell proliferation, apoptosis, and other cell processes. Furthermore, the dysregulation of miRNA expression is associated with diagnosis, progression, and response to treatments [82,83].

The majority of dysregulated miRNAs in CC are downregulated (e.g., miR-34a, miR-23b, miR-125b, miR-218, miR-497-5p, miR-146a, miR-126, miR-875-5p, and miR-155). This reduced expression has been linked to poorer overall survival outcomes. For instance, Chen et al., identified that the downregulation of miR-34a is associated with progression and metastasis in CC through the targeting of Bcl2 and c-Met [84]. Additionally, one study reported that the downregulation of miR-125b correlates with advanced tumor stage, lymph node metastasis, and shorter survival in CC patients [85].In contrast, five miRNAs were found to be upregulated (e.g., miR-133b, miR-205-5p, miR-196a, miR-21, and miR-9). The upregulation of miR-9 has been linked to oncogenic effects and is associated with higher tumor staging based on the FIGO system, lymph node metastasis, and vascular invasion [86]. Increased expression of miR-196a is correlated with shorter overall survival, and positive regulation is associated with advanced tumor stage [65]. These data are summarized in Table 3.

These findings underscore the potential of miRNAs as biomarkers in CC, providing valuable prognostic information and identifying potential therapeutic targets. The downregulation of tumor suppressor miRNAs is generally associated with poorer survival and increased tumor aggressiveness, whereas the overexpression of oncogenic miRNAs contributes to disease progression. However, despite these promising findings, the current evidence supporting the clinical application of miRNAs remains limited. Most available studies are retrospective, single-center investigations with relatively small sample sizes, and important confounding factors, including HPV genotype, disease stage, treatment history, sample type, and ethnic background, are not consistently controlled. Such heterogeneity contributes to variability in reported miRNA expression profiles and may partially explain discrepancies observed for certain miRNAs, such as miR-146a [36,87].

Beyond their diagnostic and prognostic value, accumulating evidence suggests that miRNAs may influence therapeutic response in CC. Dysregulated miRNAs have been associated with chemotherapy and radiotherapy resistance by modulating apoptosis, the DNA damage response, the epithelial–mesenchymal transition, and cancer stem cell-related pathways. Consequently, alterations in miRNA expression may affect treatment sensitivity and clinical outcomes. These observations highlight the potential of miRNAs not only as biomarkers of therapeutic response but also as promising targets for novel therapeutic strategies. Nevertheless, large-scale multicenter studies and prospective validation cohorts are still required before miRNA-based biomarkers can be routinely implemented in clinical practice [24,88].

**Table 3:** miRNAs involved in CC clinical outcome.

miRNAs	Expression	Outcome	Ref.
miR-34a	Downregulated	Shorter overall survival	[84]
miR-23b	Downregulated	Shorter overall survival	[89]
miR-125b	Downregulated	Shorter overall survival	[85]
miR-133b	Upregulated	Shorter overall survival	[90]
miR-218	Downregulated	Shorter overall survival	[91]
miR-196a	Upregulated	Shorter overall survival	[65]
miR-497-5p	Downregulated	Poor survival	[92]
miR-21	Upregulated	Shorter overall survival	[87]
miR-205-5p	Upregulated	Poor survival	[60]
miR-146a	Downregulated	Shorter overall survival	[87]
miR-424-5p	Downregulated	Poor prognosis	[93]
miR-9	Upregulated	Shorter overall survival	[86]
miR-126	Downregulated	Shorter overall survival	[94]
miR-875-5p	Downregulated	Shorter overall survival	[80]
miR-155	Downregulated	Shorter overall survival	[87]

This table summarizes the role of miRNAs in the clinical outcome of CC. The ‘miRNAs’ column lists miRNAs associated with clinical outcomes. The ‘Expression’ column indicates whether the regulation of each miRNA is upregulated or downregulated. The ‘Outcome’ column describes the clinical outcome associated with miRNA. The ‘Ref’ column cites the studies that reported these interactions.

## 5. Computational Strategies for the Identification of Differentially Expressed microRNAs in Cervical Cancer

Recent studies have demonstrated that miRNAs play a crucial role in regulating gene expression and are frequently dysregulated in this type of tumor [95]. The identification of differentially expressed miRNAs (DE-miRNAs) can provide promising biomarkers for diagnosis, prognosis, and therapeutic targets [96]. In this context, computational approaches have become fundamental for analyzing large transcriptomic datasets, enabling the precise identification of gene expression patterns associated with the development and progression of CC [97].

### 5.1. Computational Approaches for the Identification of DE-miRNAs

#### 5.1.1. Differential Expression Analysis

The identification of differentially expressed miRNAs is typically performed using robust statistical analyses of data obtained from RNA sequencing (RNA-seq) or microarrays. The main tools employed include:

DESeq2: In recent years, bioinformatics and computational biology, combined with omics and transcriptomics techniques, have played an essential role in biomedicine, particularly in the identification of biomarkers for precision medicine and drug discovery. Differential gene expression (DGE) analysis is widely used to process RNA-seq data, enabling the identification of differentially expressed genes under various experimental conditions. This approach facilitates functional enrichment analyses, providing biological insights into identified genes and aiding in the discovery of new therapeutic targets. DESeq2 is a statistical tool based on negative binomial count models, widely used to analyze RNA-seq data by estimating dispersion and variation with high precision. This method applies shrinkage techniques for fold changes and dispersion, enhancing the robustness of results, especially in studies with small sample sizes [98].

edgeR: is a Bioconductor software package designed for differential expression analysis of RNA-seq and other count-based genomic data. It employs statistical methods based on the negative binomial distribution to model count variability, making it suitable for datasets with biological replication [99].

limma: Based on Bayesian linear models, applied for the analysis of microarray and RNA-seq data, allowing efficient normalization and control of confounding variables [100].

#### 5.1.2. Integration of Multi-Omics Data

The integration of multi-omics data has significantly contributed to the identification of DE-miRNAs involved in the pathogenesis of CC. By combining transcriptomic, proteomic, and genomic data, researchers can improve the accuracy of miRNA biomarker discovery, providing valuable insights into the molecular mechanisms underlying tumor progression and treatment response.

miRTarBase and TargetScan: These databases provide experimentally validated and predicted miRNA-mRNA interactions, enabling the functional annotation of DE-miRNAs. miRTarBase compiles validated interactions through literature curation, whereas TargetScan predicts miRNA binding sites based on sequence complementarity and evolutionary conservation [101]. In CC research, these tools assist in identifying key miRNA targets implicated in oncogenic pathways, such as PI3K/AKT and TGF-β signaling [102,103].

The Cancer Genome Atlas (TCGA) and Gene Expression Omnibus (GEO): These public genomic repositories offer large-scale datasets containing transcriptomic profiles from CC and normal tissue samples. TCGA provides integrated molecular characterization, including miRNA-seq, mRNA-seq, and DNA methylation data, allowing for comprehensive cross-validation of candidate biomarkers. GEO, on the other hand, hosts microarray and sequencing datasets that enable meta-analyses across different patient cohorts, improving the reproducibility of miRNA expression studies [104].

By leveraging these multi-omics resources, researchers can enhance the identification of clinically relevant miRNAs and gain a more holistic view of their functional roles in CC progression and therapeutic resistance.

#### 5.1.3. Application of Machine Learning Algorithms

Machine learning (ML) techniques have emerged as powerful tools for analyzing transcriptomic data and uncovering hidden patterns that contribute to CC diagnosis, prognosis, and treatment stratification. ML approaches facilitate the classification of miRNA expression profiles and improve the predictive power of biomarker discovery models.

Random Forest and Support Vector Machines (SVM): These supervised learning algorithms are widely applied in cancer bioinformatics to differentiate between normal and malignant cervical tissue samples based on miRNA expression patterns. Random Forest constructs multiple decision trees and integrates their predictions to improve classification accuracy, while SVM maps expression data into a high-dimensional space to identify optimal decision boundaries for distinguishing cancerous from non-cancerous samples [105]. Studies utilizing these techniques have successfully identified miRNA signatures predictive of CC stage and patient survival [106].

Artificial Neural Networks (ANNs): Deep learning models, such as convolutional and recurrent neural networks, have demonstrated remarkable efficiency in analyzing large-scale miRNA expression datasets. ANNs can recognize non-linear relationships between miRNAs and their target genes, thereby improving the accuracy of biomarker prediction [107]. Recent studies have applied ANN models to integrate multi-omics data, achieving higher specificity and sensitivity in identifying miRNA-based prognostic markers for CC [108].

By incorporating machine learning into miRNA research, computational methods can enhance biomarker discovery, optimize patient stratification, and ultimately contribute to the development of personalized therapeutic strategies for CC treatment.

The application of computational strategies for the identification of DE-miRNAs has led to substantial advances in understanding the molecular biology of CC. The use of statistical tools for differential expression analysis, the integration of multi-omics data, and the application of machine learning have enabled the discovery of biomarkers with significant clinical potential. Future studies should focus on the functional validation of these miRNAs and the refinement of predictive approaches to enhance their clinical applicability. Although computational analyses have considerably accelerated the discovery of miRNA biomarkers in CC, translating these findings into clinically actionable tools remains challenging. Predicted miRNA–mRNA interactions often lack experimental validation, and expression signatures may vary according to HPV genotype, disease stage, sample source, and patient population. Furthermore, machine learning models are frequently developed using limited retrospective datasets, increasing the risk of overfitting and reducing external reproducibility. Future advances will depend on integrating multi-omics datasets with clinically annotated databases and automated therapeutic mapping frameworks, thereby enabling the interpretation of molecular signatures within a clinically relevant context. Importantly, prospective validation, functional studies, and standardized analytical pipelines will be required before miRNA-based panels can be reliably incorporated into precision oncology practice [109,110,111].

## 6. Controversies and Open Questions

### 6.1. Context-Dependent Functions and Conflicting Expression Patterns

Although numerous studies have identified dysregulated miRNAs in cervical cancer, their biological functions are not always consistent across different experimental and clinical contexts. A representative example is miR-146a, whose expression and biological effects appear to be highly context-dependent. HPV16 E5-mediated upregulation of miR-146a has been associated with enhanced cell adhesion and early proliferative events during HPV-driven transformation [36]. In contrast, decreased miR-146a expression in advanced cervical tumors has been linked to aggressive disease behavior and poorer survival outcomes [87]. These seemingly conflicting findings suggest that miR-146a may exert stage-specific functions throughout cervical carcinogenesis, acting differently during early HPV-induced transformation and later stages of tumor progression. Such observations highlight the complexity of miRNA-mediated regulation and underscore the importance of considering disease stage, HPV oncoprotein activity, and tumor microenvironment when interpreting miRNA expression profiles.

### 6.2. Limitations of miRNAs as Diagnostic and Prognostic Biomarkers

Although miRNAs have shown considerable promise as diagnostic, prognostic, and predictive biomarkers in CC, several challenges related to study heterogeneity, analytical methodologies, and clinical validation warrant further investigation. One major limitation is the substantial heterogeneity among studies regarding patient populations, HPV genotypes, disease stages, sample sources (tissue, plasma, serum, or cervical cytology), and analytical methodologies. Such heterogeneity often leads to considerable variation in reported biomarker performance, including differences in sensitivity, specificity, and predictive accuracy [112,113,114,115]. Furthermore, miRNA expression may be influenced by factors unrelated to cervical carcinogenesis, including inflammation, co-infections, and technical differences in sample processing [112,116]. These factors can contribute to inter-study variability and complicate the interpretation of results. In addition, the lack of standardized protocols for miRNA detection, normalization, and quantification remains an important challenge for the clinical validation and implementation of miRNA-based biomarkers.

### 6.3. Challenges in miRNA-Based Therapy

The miRNAs have emerged as a promising strategy for CC treatment due to their ability to simultaneously regulate multiple genes and signaling pathways involved in tumor progression [117,118]. Approaches based on miRNA mimics aim to restore the expression of tumor-suppressive miRNAs, whereas anti-miRNA oligonucleotides are designed to inhibit oncogenic miRNAs. Despite encouraging preclinical results, several challenges remain before miRNA-based therapies can be translated into clinical practice [117].

One of the major obstacles is the efficient and selective delivery of therapeutic miRNAs to target tissues. Unencapsulated miRNAs are highly susceptible to degradation by circulating nucleases and often exhibit limited cellular uptake, necessitating specialized delivery systems such as nanoparticles, liposomes, viral vectors, or extracellular vesicles. However, these platforms may have limitations in stability, biodistribution, immunogenicity, and toxicity [119]. Another important concern is the occurrence of off-target effects. Because a single miRNA can regulate multiple mRNA targets, therapeutic modulation may unintentionally affect unrelated cellular pathways, potentially leading to unpredictable biological consequences [120].

Taken together, these challenges underscore the importance of improving delivery strategies, enhancing target specificity, and establishing robust preclinical validation frameworks to facilitate the safe and effective clinical application of miRNA-based therapeutics in cervical cancer.

## 7. Conclusions

In conclusion, miRNAs play a pivotal role in the regulation of key oncogenic processes in HPV-driven CC, including cell proliferation, apoptosis, immune evasion, and metastatic spread. This review highlights the intricate interactions between HPV onco-proteins and miRNA-mediated modulation of critical signaling pathways. However, despite significant advances in our understanding of these mechanisms, several important questions remain unanswered. Notably, most available data are derived from in vitro studies or small clinical cohorts, and there is a need for large-scale, integrative studies to validate miRNAs as robust diagnostic or prognostic biomarkers. Moreover, the therapeutic modulation of miRNAs, either through mimic or inhibitor strategies, has shown promise in preclinical models but requires further translational research and clinical trial validation. Future investigations should focus on standardizing miRNA profiling techniques, clarifying their role in treatment resistance, and exploring the potential of miRNA-based therapeutics in combination with existing treatment modalities. A deeper understanding of the molecular networks regulated by miRNAs may ultimately contribute to the development of more precise diagnostic tools and personalized therapeutic strategies for patients with CC.

## Figures and Tables

**Table 1:** Summary of HPV Oncoproteins (E5, E6, and E7) and Their Regulation of miRNAs in CC.

Protein	miRNAs	miRNA Regulation	Target	Cell Process	Ref.
E5	miR-146a	Up	ZNF813	Cell adhesion and cell cycle	[36]
E5	miR-203	Down	p63	Cell junction, cell migration, and cell motility	[36]
E5	miR-324-5p	Down	CDH2, CTNNB1	Transendothelial migration	[36]
E6	miR-409-3p	Down	PI3K/AKT	Proliferation and migration	[37]
E6	miR-143	Up	HIF-1a, PD-L1	Immune system	[38]
E7	miR-27b	Up	PLK2	Cell proliferation, invasion and apoptosis	[39]
E7	miR-21	Up	No identified	Cell growth, proliferation and invasion	[40]
E6/E7	miR-17-5p, miR-186-5p, miR-378a-3p, miR-378f, miR-629-5p, and miR-7-5p (intracellular)	Down	No identified	Cell proliferation and apoptosis	[41]
E6/E7	miR-143-3p, miR-23a-3p, miR-23b-3p, and miR-27b-3p (intracellular)	Up	No identified	Cell proliferation and apoptosis	[41]
E6/E7	let-7d-5p, miR-20a-5p, miR-378a-3p, miR-423-3p, miR-7-5p, miR-92a-3p (exosomal)	Down	p21	Cell proliferation and apoptosis	[41]
E6/E7	miR-21-5p (exosomal)	Up	p21	Cell proliferation and apoptosis	[41]
E6/E7	miR-142-5p	Down	PD-L1	Immune system	[42]
E6/E7	miR-331-3p	Up	NRP2	Cell proliferation, cell cycle, apoptosis	[43]
E6/E7	miR-199b-5p and miR-190a-5p	Down	No identified	Cell proliferation	[44]
E5/E6/E7	miR-148a-3p	Down	No identified	Cell proliferation	

This table summarizes the role of HPV oncoproteins (E5, E6, and E7) in regulating microRNAs (miRNAs) involved in CC progression. The “Protein” column lists the HPV oncoproteins responsible for miRNA regulation. The “miRNAs” column shows the specific miRNAs affected by these oncoproteins. The “miRNA Regulation” column indicates whether the miRNA is upregulated (increased) or downregulated (decreased). The “Target” column identifies the key molecular targets of the regulated miRNAs. The “Cell Process” column describes the biological processes influenced, such as cell proliferation, migration, apoptosis, and immune evasion. The “Ref.” column references the studies that reported these interactions.

**Table 2:** Overview of miRNAs, their Targets, associated signaling pathways, and hallmarks of cancer.

miRNA	Target	Signaling Pathways	Hallmarks of Cancer	Ref.
miR-34a	p18Ink4c	E6/p53	Senescent cells, evading growth suppressors and resisting cell death	[50,51]
miR-23b	uPA	E6/p53	Activating invasion & metastasis	[53]
miR-375	E6 and E7	E6/p53 and E7/pRb	senescent cells and resisting cell death	[61]
miR-129-5p	SP1	E6/p53 and E7/pRb	Sustaining proliferative signaling	[63]
miR-92	PTEN	PI3K/AKT	Sustaining proliferative signaling, activating invasion & metastasis and resisting cell death	[54]
Cluster miR-302 and miR-367	Cyclin D1, AKT1, and p27	PI3K/AKT	Sustaining proliferative signaling	[64]
miR-196a	FOXO1 and p27	PI3K/AKT	Sustaining proliferative signaling	[65]
miR-125b	PIK3CD	PI3K/AKT/mTOR	Sustaining proliferative signaling and resisting cell death	[55]
miR-133b	MST2, CDC42 and RHOA	AKT/ERK	Sustaining proliferative signaling	[59]
miR-218	RICTOR	AKT/mTOR	Sustaining proliferative signaling and resisting cell death	[66]
miR-23b-3p	c-Met	FAK/AKT/ERK	Sustaining proliferative signaling and activating invasion & metastasis	[67]
miR-21	PTEN	FAK/AKT/ERK	Sustaining proliferative signaling and activating invasion & metastasis	[58]
miR-7	FAK	FAK/AKT/ERK	Sustaining proliferative signaling and activating invasion & metastasis	[68]
miR-497-5p	MAPK1	MAPK	Sustaining proliferative signaling and activating invasion & metastasis	[69]
miR-454-3p	TRIM3	MAPK	Evading growth suppressors and resisting cell death	[70]
miR-135a	SIAH1	Wnt/β-catenin	Activating invasion & metastasis and sustaining proliferative signaling	[71]
miR-205-5p	AXIN2	Wnt/β-catenin	Resisting cell death and sustaining proliferative signaling	[60]
miR-146a	CTNNB1	Wnt	Activating invasion & metastasis and sustaining proliferative signaling	[48]
miR-4524b-5p	WTX	Wnt/β-catenin	Activating invasion & metastasis	[72]
miR-424-5p	KDM5B	Notch	Sustaining proliferative signaling and resisting cell death	[73]
miR-34a	Notch1 and Jagged1	Notch	Activating invasion & metastasis	[52]
miR-628-5p	Jagged1	Notch	Sustaining proliferative signaling and resisting cell death	[74]
miR-130a-3p	DLL1 e WNT10A	Notch/Wnt	Activating invasion & metastasis and sustaining proliferative signaling	[75]
miR-506	Gli3	Hedgehog	Sustaining proliferative signaling, evading growth suppressors and resisting cell death	[76]
miR-9	IL-6	JAK/STAT	Sustaining proliferative signaling, activating invasion & metastasis and unlocking phenotypic plasticity	[77]
miR-126	MMP2, MMP9 and ZEB1	JAK/STAT	Sustaining proliferative signaling and activating invasion & metastasis	[78]
miR-211	ZEB1	JAK/STAT	Sustaining proliferative signaling and activating invasion & metastasis	[79]
miR-875-5p	EGFR	EGFR	Sustaining proliferative signaling and activating invasion & metastasis	[80]
miR-155	EGF	EGFR	Sustaining proliferative signaling, activating invasion & metastasis	[81]

This table provides an overview of miRNAs involved in CC. The ‘miRNAs’ column lists miRNAs associated with CC. The ‘Target’ column identifies the primary molecular targets regulated by these miRNAs. The ‘Signaling pathways’ column describes the signaling pathways in which each miRNA is involved. The ‘Hallmarks of Cancer’ column indicates the specific hallmarks of cancer associated with each miRNA. The ‘Ref’ column cites the studies that reported these interactions.

## Data Availability

No new data were created or analyzed in this study. Data sharing is not applicable to this article.

## Abbreviations

The following abbreviations are used in this manuscript:

AKT	Protein kinase B
AMER1	APC membrane recruitment protein 1
ANNs	Artificial Neural Networks
AXIN2	Axis Inhibition Protein 2
CC	Cervical Cancer
CDC42	Cell division control protein 42 homolog
CIN	Cervical intraepithelial neoplasia
c-MET	C-Mesenchymal–Epithelial Transition Factor
CTNNB1	Catenin beta 1
DE-miRNAs	Differentially expressed miRNAs
DGCR8	DiGeorge Critical Region 8
DGE	Differential gene expression
DISC	Death-Inducing Signaling Complex
DLL1	Delta-like notch 1 ligand
EGF	Epidermal Growth Factor
EGFR	Epidermal Growth Factor Receptor
EMT	Epithelial–mesenchymal transition
ERK	Extracellular signal-regulated kinase
FAK	Focal Adhesion Kinase
GEO	Gene Expression Omnibus
GLI3	GLI family zinc finger 3
HIF-1α	Hypoxia-Inducible Factor 1-alpha
HPV	Human Papillomavirus
hr-HPV	High-risk Human Papillomavirus
IL-6	Interleukin-6
KDM5B	Lysine demethylase 5B
lr-HPV	Low-risk Human Papillomavirus
MAPK1	Mitogen-Activated Protein Kinase 1
MHC I	Major Histocompatibility Complex class I
miRNAs	MicroRNAs
ML	Machine learning
MMP2	Matrix Metalloproteinase-2
MMP9	Matrix Metalloproteinase-9
MST2	Mammalian sterile 20-like kinase 2
NRP2	Neuropilin 2
OncomiRs	Oncogenic miRNAs
P18INK4c	p18 inhibitor of cyclin-dependent kinase 4c
PD-L1	Programmed Death-Ligand 1
PI3K	Phosphoinositide-3 kinase
PIK3CD	Phosphoinositide-3 kinase delta
PLK2	Polo-like kinase2
pRb	Retinoblastoma protein
PTEN	Phosphatase and Tensin Homolog
RHOA	Ras homolog gene family member A
RNA	Ribonucleic acid
RNA-seq	RNA sequencing
SP1	Specificity Protein 1
SVM	Random Forest and Support Vector Machines
TCGA	The Cancer Genome Atlas
TRIM3	Tripartite motif-containing 3
ts-miRs	Tumor-suppressor genes
UBE2I	Ubiquitin-conjugating enzyme E2I
uPA	Urokinase-type plasminogen activator
WNT10A	Wnt family member 10a
WTX	Wilms tumor gene on the X chromosome
ZEB1	Zinc finger E-box binding homeobox 1
ZNF813	Zinc finger protein
